# Supplementary figures and images for: Buscando Luciérnagas: findings on Mexican fireflies from an 8-year virtual citizen science project
Source: PeerJ. 2024 Sep 18;12:e18141. doi: 10.7717/peerj.18141 (PMC11416087; doi:10.7717/peerj.18141)

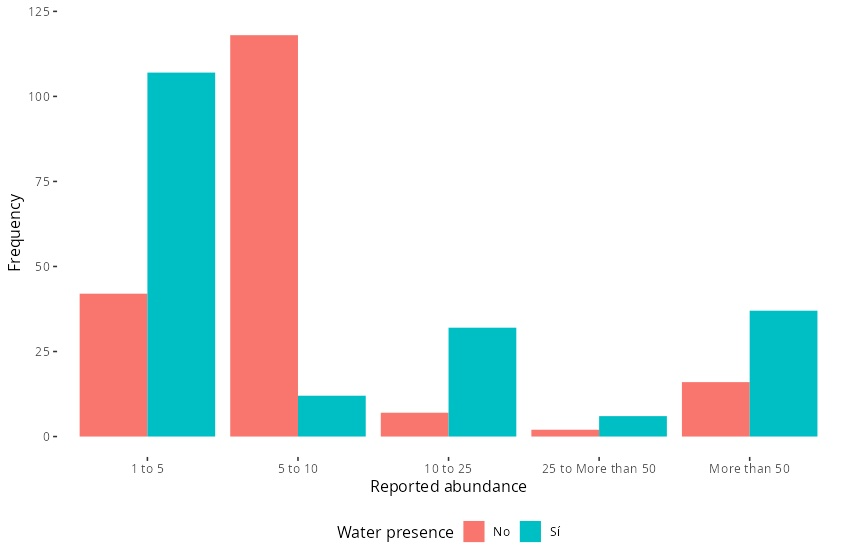

Supplement: Supplemental Information 2 — Number of reports per firefly abundance category related with water presence [file peerj-12-18141-s002.png]

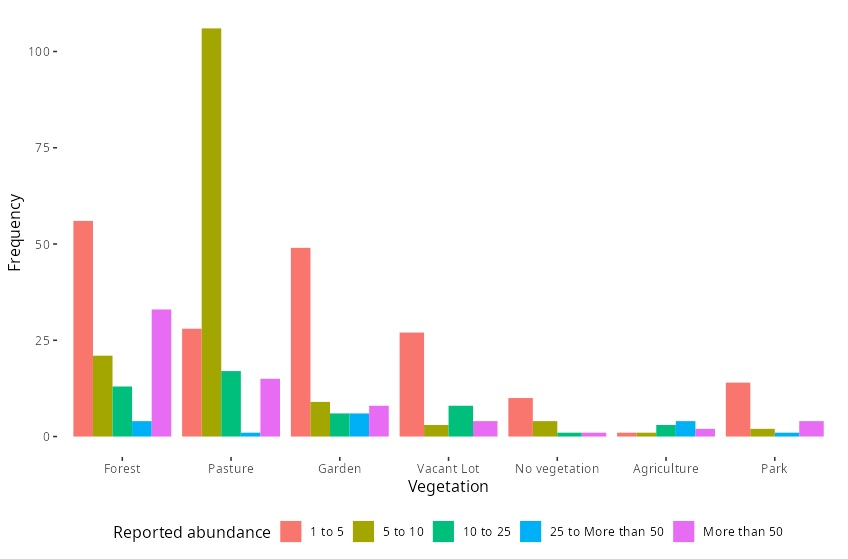

Supplement: Supplemental Information 3 — Number of reports per vegetation type considering the different firefly abundance categories [file peerj-12-18141-s003.png]
